# The Role of Gamma-Delta T Cells in Diseases of the Central Nervous System

**DOI:** 10.3389/fimmu.2020.580304

**Published:** 2020-10-23

**Authors:** Jin Wo, Feng Zhang, Zhizhong Li, Chenghong Sun, Wencai Zhang, Guodong Sun

**Affiliations:** ^1^ Department of Orthopedics, First Affiliated Hospital, Jinan University, Guangzhou, China; ^2^ Intensive Care Unit, First Affiliated Hospital, Jinan University, Guangzhou, China; ^3^ State Key Laboratory of Generic Manufacture Technology of Chinese Traditional Medicine, Linyi, China

**Keywords:** γδ T cell, Th17 cell, cytokines, inflammation, central nervous system, IL-17

## Abstract

Gamma-delta (γδ) T cells are a subset of T cells that promote the inflammatory responses of lymphoid and myeloid lineages, and are especially vital to the initial inflammatory and immune responses. Given the capability to connect crux inflammations of adaptive and innate immunity, γδ T cells are responsive to multiple molecular cues and can acquire the capacity to induce various cytokines, such as GM-CSF, IL-4, IL-17, IL-21, IL-22, and IFN-γ. Nevertheless, the exact mechanisms responsible for γδ T cell proinflammatory functions remain poorly understood, particularly in the context of the central nervous system (CNS) diseases. CNS disease, usually leading to irreversible cognitive and physical disability, is becoming a worldwide public health problem. Here, we offer a review of the neuro-inflammatory and immune functions of γδ T cells, intending to understand their roles in CNS diseases, which may be crucial for the development of novel clinical applications.

## Introduction

Together, gamma-delta (γδ) and alpha-beta (αβ) T cells represent two different T cell lineages that have been defined by their expression of αβ or γδ T cell receptors (TCRs) (1). Although γδ T cells share many effector capabilities with αβ T cells (for example, cytotoxicity and cytokine production), the lineages exhibit different biological properties, such as thymic-dependent or -independent development, major histocompatibility complex (MHC) restriction, and recognition of soluble protein and non-protein antigens of endogenous origin (2–5).

Unlike αβ T cells, γδ T cells are a relatively minor subset of T lymphocytes in the peripheral blood (PB), comprising only 1–5% of lymphocytes circulating (6). However, γδ T cells are abundant at barrier sites such as the skin, gut, lung, and reproductive tract; up to 20% of intraepithelial lymphocytes in the human colon express the γδ TCRs (7).

γδ T cells are divided according to the type of Vγ and Vδ chain they express at the TCRs. Concerning the Vγ chains, a unique feature of murine γδ T cells is the preferential expression of different Vγ segments in different tissues. For example, Vγ5^+^ γδ T cells are present in the skin, Vγ7^+^ γδ T cells lie in the intestinal, Vγ6^+^ γδ T cells localize to the reproductive mucosa, and Vγ1^+^ or Vγ4^+^ γδ T cells are found in secondary lymphoid organs (8, 9). The previous studies related to human γδ T cells have identified Vγ9 as the most frequently used Vγ chain in the PB (10). Vγ9 chain associates with Vδ2 in most cases, defining a Vγ9Vδ2 T cell population (account for 50–95% of γδ T cells in the PB) that is unique to humans and other primates (11, 12).

Vγ9Vδ2 T cells are known to identify microbe-derived [HMBPP, (E)-4-hydroxy-3methyl-but-2-enyl pyrophosphate] and host-derived (IPP, isopentenyl pyrophosphate) phosphorylated metabolites originating from the isoprenoid metabolic mevalonate and non-mevalonate pathways, through association with butyrophilin 3A1 (BTN3A1) and BTN3A2 (13–16). Moreover, Vδ1^+^ γδ T cells frequently coexpress functional receptors of innate immune cells, such as activating natural killer (NK) receptors such as NKG2D (17–20). It includes MHC class I polypeptide-related chains (MIC) A and B, and UL16 binding proteins (ULBP) (21–24). Although first described for Vδ1^+^ γδ T cells, interactions of the ULBP and MIC-A/B molecules with NKG2D are now recognized to stimulate Vδ2^+^ γδ T cells (21, 22). Besides, Vδ1^+^ γδ T cells recognize lipids and glycolipids presented by CD1 molecules (25, 26). Furthermore, both Vδ1^+^ and Vδ2^+^ γδ T cells are activated by heat shock proteins (HSP) (27–29).

Recently, some discrete population of T cells that coexpressed αβ-γδ TCRs and Vγ-Cβ TCRs have been identified (30, 31). Among them, the αβ-γδ T cells protected against infection by licensing encephalitogenic Th17 cells, triggered inflammatory and immune in the central nervous system (CNS). Moreover, our research group found that, in addition to diseases of the CNS, such as multiple sclerosis (MS) and stroke, immune responses induced by γδ T cells are also critically implicated in neuroinflammation associated with spinal cord injury (SCI) (32–34). These findings raise significant questions concerning the inflammatory and immune functions of γδ T cells in CNS disease that have yet to be addressed (35–37). CNS disease, which can result in irreversible sensory, motor, and autonomic impairments, is a severe health problem worldwide. As a central pathological process in CNS diseases, the inflammatory response is vital to clinical prognosis. Here, we provide a review of recent advances in the understanding of γδ T cells with relevance to their inflammatory and immune roles in CNS disease, which suggest potential approaches for future treatment of CNS diseases (**Table 1**).

**Table 1:** The role of γδ T cells in CNS diseases.

Disease	Species	γδ T subset	Tissue/organ	Cytokine/antigen	Conclusion	References
MS	Human	Vγ9/Vδ1/Vδ2	Brain	HSP60/HSP90	Detrimental	(27)
MS	Human	Vγ2/Vδ1/Vδ2	PB/CSF	HSP70	Detrimental	(28)
MS	Human	Vδ1/Vδ2	PB/CSF	–	Detrimental	(81)
MS	Human	–	PB/CSF	IL-17	Detrimental	(82)
MS	Human	–	Brain/CSF	HSP72	Detrimental	(87)
MS	Human	Vδ1	PB	IFN-γ	Detrimental	(90)
MS	Human	–	Brain	HSP65/HSP90	Detrimental	(93)
EAE	Mouse	–	Spinal cord	HSP60	Detrimental	(80)
EAE	Mouse	–	Brain/Spinal cord	IL-12	Detrimental	(83)
EAE	Mouse	–	Spinal cord	–	Detrimental	(84)
EAE	Mouse	–	Spinal cord	MIP-1α/MCP-1	Detrimental	(86)
EAE	Mouse	Vγ4/Vγ5/Vδ6	Brain	IL-17/IL-21/IL-22	Detrimental	(40)
EAE	Mouse	Vγ4	PB	IL-17	Detrimental	(92)
EAE	Mouse	–	Spleen	IL-15	Detrimental	(94)
EAE	Mouse	–	Brain/Spinal cord	–	Protective	(95)
Stroke	Mouse	–	Brain	IL-17	Detrimental	(99)
Stroke	Human/Mouse	–	Brain	IL-17	Detrimental	(101)
Stroke	Mouse	Vγ6	Brain	IL-17/TNF-α	Detrimental	(103)
Stroke	Mouse	–	Brain/PB	IL-17	Protective	(124)
PVL	Human/Mouse	–	Brain	IL-17F/IL-22	Detrimental	(104)
WNV infection	Mouse	–	Brain/Spleen/PB	–	Protective	(107)
HSV-1 infection	Mouse	–	Brain/Trigeminal ganglia	–	Protective	(108)
Bacterial meningitis	Human	Vγ9/Vδ2	PB/CSF	IL-17	Protective	(111)
Brain abscess	Mouse	–	Brain	IL-17	Protective	(112)
Brain abscess	Mouse	–	Brain	IL-17	Protective	(115)
TBI	Mouse	–	Brain/PB	–	Detrimental	(119)
SCI	Mouse	Vγ4	Spinal cord/CSF	IFN-γ/TNF-α	Detrimental	(34)
Epilepsy	Human	–	Brain	IL-17/GM-CSF	Detrimental	(125)
RE	Human	Vδ1	Brain	–	Detrimental	(126)

MS, multiple sclerosis; HSP, heat shock protein; PB, peripheral blood; CSF, cerebrospinal fluid; EAE, experimental autoimmune encephalomyelitis; MIP, macrophage-inflammatory protein; MCP, monocyte chemoattractant protein; PVL, periventricular leukomalacia; WNV, west nile virus; HSV, herpes simplex virus; TBI, traumatic brain injury; SCI, spinal cord injury; RE, rasmussen encephalitis.

## Proinflammatory Cytokines Induced by γδ T Cells in the CNS

Activation and development of γδ T cells promoting CNS inflammation are chiefly mediated by dendritic cells (DCs). The immunostimulatory component induces IL-1β, IL-6, IL-18, and IL-23 by DCs *via* caspase-1 and inflammasome complex. γδ T cells secrete IL-17 in response to IL-1β, IL-18, and IL-23 in the absence of TCR (38–40). During this process, the retinoid-related orphan receptor (ROR) -γt and IL-7 coordinate the B and T lymphocyte attenuator (BTLA) expression, thus regulating γδ T cell inflammatory responses (41–44). Moreover, Shibata et al. demonstrated that signal transducer and activator of transcription 3 (STAT3) is dispensable for the development of IL-17-producing γδ T (γδT17) cells (45). Also, IL-23-activated γδ T cells suppress the factor forkhead box P3^+^ (Foxp3) -expressing Treg cells conversion, as well as promoting effector T (Te) cells response (46, 47). The capacity of γδ T cells to produce a burst of IL-17 in the absence of activated αβ T cells is crucial for the initiation of CNS inflammation (48).

**Figure 1:**
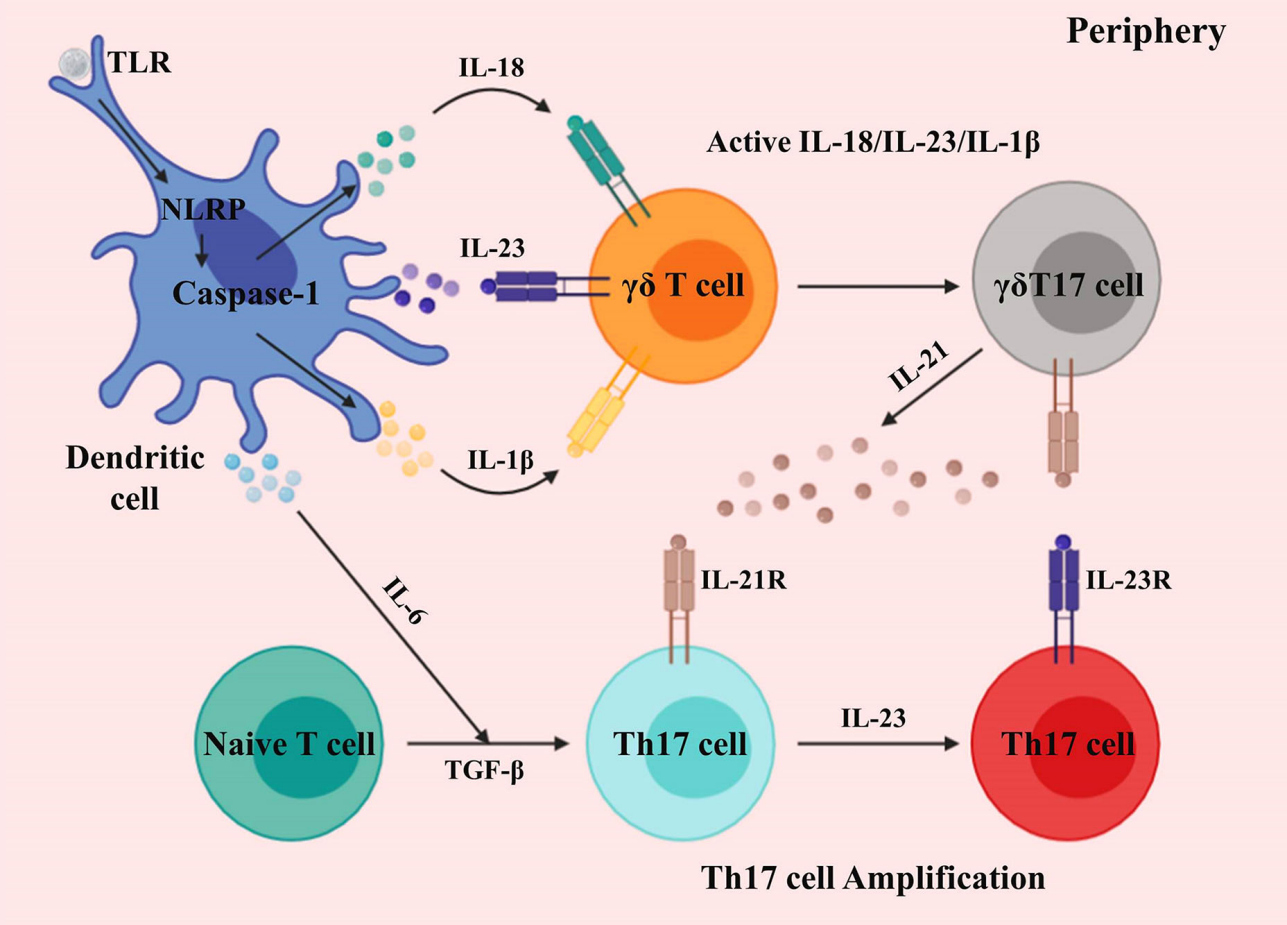
Activation and development of γδ T cells in the periphery. Differentiated dendritic cells and macrophages generate proinflammatory cytokines *via* toll-like and NOD-like receptors. γδ T cells sense IL-1β, IL-18, and IL-23, producing an initial burst of IL-17. The differentiation of Th17 cells is induced by IL-6 and TGF-β. γδT17 cells secrete IL-21, which further amplifies their proliferation, and also that of Th17 cells.

Activated DCs also promotes the induction of other proinflammatory cytokines from γδ T cells, such as granulocyte-macrophage colony-stimulating factor (GM-CSF), IL-21, and IL-22 (30, 40) (**Figure 1**). While IL-17A, IL-17F, and IL-22 are prominently expressed in CNS inflammation, they may only marginally contribute to disease development (49–51); however, McGinley et al. recently demonstrated that IL-17 might recruit IL-1β-secreting myeloid cells that prime pathogenic γδ T cells in CNS inflammation (52).

Different from γδ T cells, which can produce IL-17 in response to cytokine (IL-1β, IL-18, and IL-23) signals alone, in the absence of primary (TCR) and secondary (costimulation) signals, IL-17-producing T helper (Th17) cells require primary, secondary, and cytokine (IL-6 and TGF-β) signals to generate IL-17 (40) (**Figure 1**). Seminal studies demonstrated that IL-6 and TGF-β induce Th17 cell differentiation, in which TGF-β is critical for T cells to differentiate into Foxp3^+^ Treg or Th17 cells (53–58). Moreover, TGF-β is also critical to γδT17 cells (59). Besides, IL-21 is induced by IL-6 in Th17 cells, which establishes a feed-forward loop to support Th17 cell amplification, in which STAT3 and ROR-γt mediate lineage specification (54, 55, 60–63).

During this process, IL-23 acts as a maturation factor for Th17 cells, and both IL-23 and IL-21 can induce IL-17 expression independently of IL-6 (55, 64–66). Therefore, mice lacking IL-23 are resistant to Th17-mediated CNS inflammation (46). To demonstrate the role of IL-23, Awasthi et al. substituted the green fluorescent protein for the intracellular domain of IL-23R, to generate a “knock-in” mouse, which demonstrated that IL-23 is crucial for Th17 cell function (67). IL-23 created a positive feedback loop, whereby GM-CSF secreted by Th17 cells induced the generation of IL-23 (68, 69).

Alongside IL-17, GM-CSF is also essential for CNS inflammation. Further, the activation of the microglial cell, but not macrophage in the periphery, is a GM-CSF-dependent process (70). El-Behi et al. demonstrated that GM-CSF neutralization attenuated CNS inflammation (68). Although both IL-12 and IL-23 can induce Te cells to generate GM-CSF, IL-23 is crucially required for GM-SCF generation (69, 71). In addition to DCs and Th17 cells, γδ T cells generate large amounts of GM-CSF, resulting in neuroinflammation (72).

## γδ T Cells in CNS Diseases

### Multiple Sclerosis and Experimental Autoimmune Encephalomyelitis

MS is a chronic inflammatory demyelinating CNS disease, resulting in progressive cognitive, sensory, and motor disorders. Experimental autoimmune encephalomyelitis (EAE), a murine MS model, is used to research the proinflammatory mechanism underlying CNS (73). Before the discovery of Th17 cells, IFN-γ-producing Th1 cells were considered the primary pathogenic cell inducing MS and EAE, which puzzled immunologists for many years, since both IFN-γ^−/−^ and IFN-γR^−/−^ mice enhanced EAE development (74–77). Besides, deficiencies of IL-12 and IL-12R, which are critical to the development of Th1 cells, also exhibited exacerbated EAE (64). Together, findings to date indicate that Th1 cells are not the initial T cell involved in EAE. IL-12 and IFN-γ (Th1-associated molecules) negatively regulate tissue inflammation in EAE (78). Nonetheless, Th1 cells are vital to EAE, as they are detected in active EAE.

**Figure 2:**
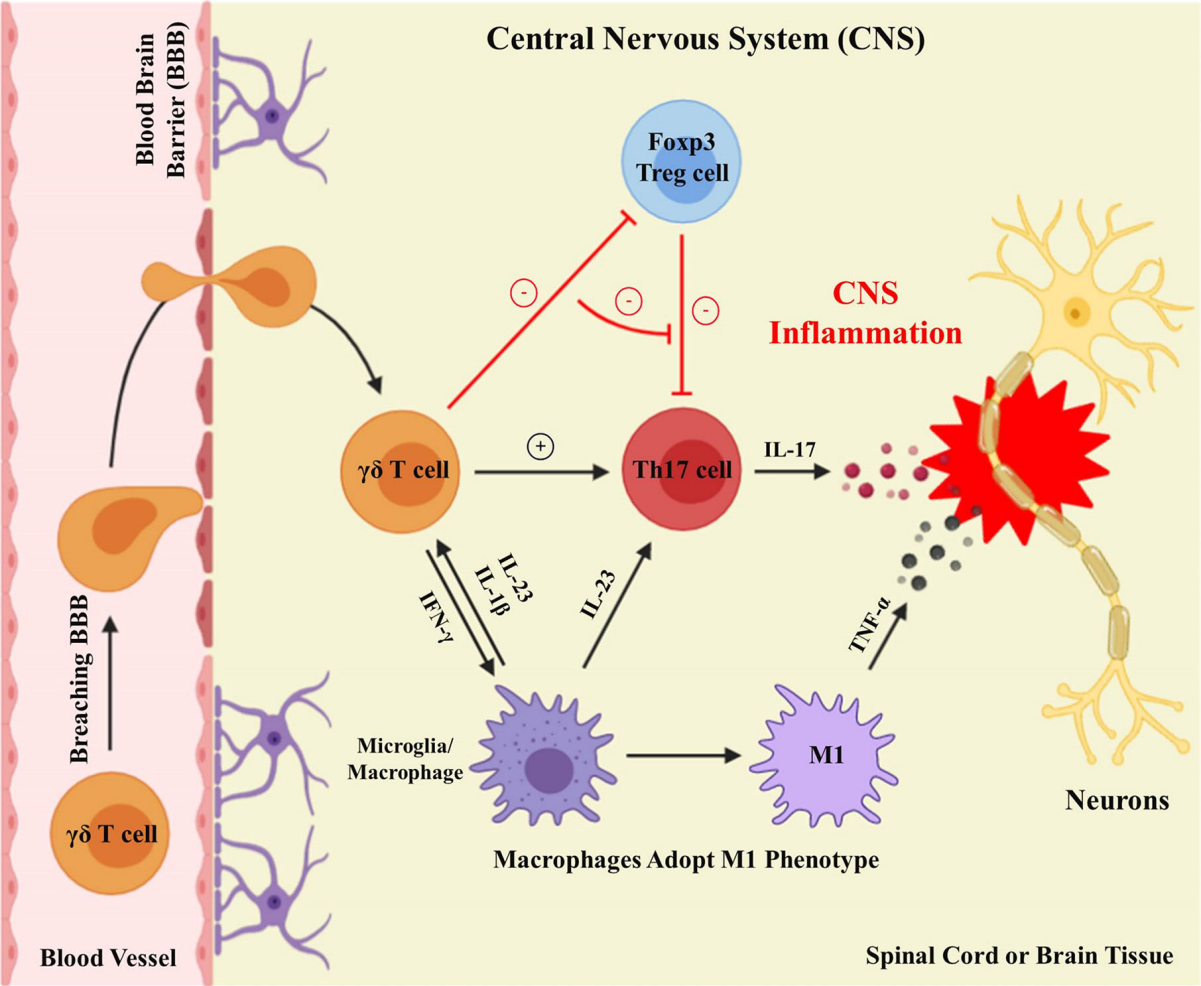
γδ T cells execute proinflammatory functions in the CNS. Activated γδ T cells breach the blood-brain barrier to carry out proinflammatory functions in the CNS. Differentiated microglia/macrophages secrete IL-23 within the CNS to facilitate the production of γδ T cells and Th17 cells. γδ T cells result in CNS inflammation by improving Th17 cell effector functions, restraining Tregs cell suppressive functions, and generating IFN-γ, to induce M1 phenotype macrophages secreting TNF-α.

Subsequently, the identification of IL-23 and Th17 cells partly worked out this issue (61, 65) (**Figure 2**). IL-23p40^−/−^ and IL-23p19^−/−^ mouse strains are both resistant to EAE (64). The depletion of Th17 cells or IL-17 resulted in reduced EAE severity (78). Although Th17 cells are thought to be the major mediators of EAE, γδT cells can also be a potent producer of IL-17, and are dominant over Th17 cells in CNS inflammation (79). Several researchers demonstrated that γδ T cells are frequently present in the peripheral blood (PB) and cerebrospinal fluid (CSF) of MS patients, as well as in the brains of mice with EAE (27, 28, 80–82). During the chronic and acute phases of EAE, the absence of γδ T cells notably reduces the CNS inflammation, suggesting that γδ T cells are significant in EAE, and their inflammatory mobilization is related to the pathogenesis of CNS autoimmunity (83–87). Indeed, an enormous population of CD4^+^ T cells (IL-17 and IFN-γ double-positive) is observed at the peak of EAE (88). Using a fate-tracking system, 5–10% of γδT17 cells were shown to express IFN-γ in the CNS, indicating that IL-17-IFN-γ-γδ T cells might be consequential intermediates in EAE pathogenesis (89).

Moreover, Vγ4^+^ γδ T cells were identified as the major γδT17 cells in EAE, while Vγ5^+^ and Vδ6^+^ γδ T cells were present (40). Vδ1^+^, Vδ2^+^, and Vγ9^+^ γδ T cells were also observed in acute demyelinating plaques of MS patients (27, 90). Besides, the biological drugs designed to suppress the activity of γδ T cells, such as fingolimod (FTY720) and Natalizumab, partly contribute to the clinical therapeutic effects of MS. (90–92). Further, HSP60 and HSP90 compared with normal CNS tissues are overexpressed in MS plaques, while Selmaj et al. showed the colocalization of HSP65 and γδ T cells in immature oligodendrocytes in MS lesions (27, 80, 93). The multitude of γδ T cells collected from MS patients proliferated in response to HSP70, but not to HSP65, revealing that HSPs may be the antigens responsible for promoting the γδ T cells proliferation (28). CNS inflammation is associated with altered expression of HSPs, which may function as targets in the development of the chronic disease. Interestingly, IL-15-producing γδ T (γδT15) cells, another subset of γδ T cell, were recently discovered; however, whether these cells produce other proinflammatory cytokines in EAE is not well elucidated (94).

In contrast to the above observations, γδ T cells are also reported have a protective function in EAE. Ponomarev et al. reported that γδ T cells of wild-type (WT) reconstitute γδ T cell^−/−^ mice, but not FasL dysfunctional γδ T cells, diminishing inflammation in EAE (95). These findings suggest that the γδ T cell-mediated Fas/FasL-induced T cells apoptosis regulates CNS inflammation. Indeed, the mechanism by which γδ T cells regulate proinflammatory chemokine and cytokine expression in CNS, as well as infiltrating cell heterogeneity, warrant detailed investigation.

### Ischemic Brain Injury

The main consequence of ischemic brain injury is manifested as the CNS tissue necrosis, due to the loss of nutrition. The tissue necrosis leads to a secondary inflammation, involving the accumulation of specific immune cells, especially neutrophils, macrophages, and T cells, which is a critical factor to the entire pathophysiology (96, 97).

IL-17 has a specific role in the delayed phase of the ischemic brain injury inflammatory cascade (98). Shichita et al. demonstrated that γδT17 cells play a significant role during late-stage ischemic brain injury, and that they, rather Th17 cells, are (surprisingly) the major origin of IL-17 (99). Moreover, increased IL-17 levels are present in the PB of patients who have suffered a stroke, relative to healthy individuals (100). IL-23, generated by macrophages or monocytes from stroke initiation, is an essential contributor for inducing IL-17 by γδ T cells during the delayed phase of encephalic ischemia. Thereby, IL-23p19^−/−^ mice illustrated a diminishment in infarct extent only 1 day after the ischemic injury, whereas IL-17 deficiency led to reduced infarct size after 4 days. Long-term, deficiencies of IL-17 and IL23 demonstrated obviously diminished CNS injury, relative to WT, or even IFN-γ^−/−^, mice (99). Gelderblom et al. demonstrated that injection of IL-17-neutralizing antibody to mice within the post-stroke 3h could reduce infarct volume and improve disease prognosis after 3 days (101).

Overall, the available evidence demonstrates that γδ T cells are the main source of IL-17. The mechanism of antigen-independent T cell activation post-stroke remains unclear; however, it has been owed chiefly to γδ T cells. Nevertheless, Kleinschnitz et al. demonstrated that γδ T cell-deficient mice remain susceptible to ischemic insult, indicating an extra function for other immune cells in ischemic brain injury. Furthermore, the fact that transgenic-TCR mice are susceptible to stroke implies that, besides γδ T cells, Th17 cells may also have a prominent role in stroke, whereas the precise function of Th17 cells in inducing stroke is not exact (102).

In addition, astrocytes can respond to IL-17 and promote stroke induction and development (101). For instance, IL-17 produced by γδ T cells and TNF-α secreted by macrophages act synergistically on astrocytes, by inducing the expression of CXCL1, a neutrophil chemoattractant (101, 103). Recently, periventricular leukomalacia (PVL), a distinctive form of brain injury in premature infants, was demonstrated to be caused by developmental immaturity of the cerebral vasculature in mid to late gestational age, and large numbers of γδ T cells were observed in postmortem brains from preterm infants (104). Although there were increased IL-17 and IL-22 in mouse brains after injury, neither cytokine contributes to preterm brain injury (104).

In summary, γδ T cells and IL-17 have essential roles in ischemic brain injury. Hence, γδ T cells and IL-17 should be considered potential therapeutic targets to decrease secondary inflammation after ischemic brain injury (105, 106).

### Central Nervous System Infection

The CNS infections commonly lead to the disruption of the blood-brain barrier (BBB) protectiveness and subsequent tissue inflammation; however, inflammation is also crucial to CNS immunity, as reduced γδ T cell expansion leads to increased host vulnerability to viral infection (107, 108). For example, MS patients treated with Natalizumab, a monoclonal antibody against α4-integrin, undergo fatal viral infections, due to the immune cells fail to infiltrate the CNS and eliminate the infection (109).

In contrast to viral infection, there are some (although limited) researches involving the function of γδ T cells in models of CNS bacterial infection (110). For example, children with bacterial meningitis exhibit high γδ T cell fractions in the CSF (111). Nichols et al. suggested that the γδ T cell was an alternative pathway available to respond to Grampositive bacteria CNS infection. They found that TLR2^−/−^ brain abscess mice (TLR2 is a critical receptor for eliciting responses to Grampositive bacteria) were detected elevated IL-17, and γδ T cells were the source of IL-17 (112–114). Similarly, IL-17R signaling regulates γδ T cell infiltration, as well as bacterial clearance, during S. aureus-induced brain abscess formation (115). Also, IL-17 expression is augmented in the CNS of mice infected with Toxoplasma gondii (116). An increased percentage of γδT17 cells was observed in the PB and lesion in children with bacterial meningitis, and the condition was reversed after antibacterial therapy (111). High levels of IL-17 can also be detected in the abscess formation of humans; however, such researches are only associated, since no direct evidence can be demonstrated (117). Nevertheless, evidence for the involvement of γδ T cells in any CNS infection is sparse, and more studies are needed to establish a relation between γδ T cells and CNS infections.

### Central Nervous System Traumatic Diseases

Immune responses and neuroinflammation involving γδ T cells are also critically involved in CNS traumatic diseases. Diseases resulting from CNS trauma usually involve irreversible sensory, motor, and autonomic impairments (118). Peripheral immune mechanisms establishment is related to the pathological processes of traumatic brain injury (TBI). Richard et al. found that CD4+ and CD8+ T cells, Tregs, and γδ T cells, increased in number within 24 h after TBI (119).

Further, recent results from our laboratory found that γδ T cells, particularly Vγ4^+^ γδ T cells, exert a detrimental role in SCI, probably by providing an important origin of IFN-γ, which induces macrophages to adopt the M1 phenotype, with increased secretion of inflammatory cytokines, such as TNF-α (34) (**Figure 2**). Moreover, one significant discovery from our studies was that bone marrow-derived macrophages (BMDMs) respond to IFN-γ. This was supported by two sets of findings. First, IFN-γR^−/−^ mice, chimeras with IFN-γR^−/−^ bone marrow, and mice receiving adoptively transferred IFN-γR^−/−^ peritoneal macrophages, all showed similar recovery following SCI. Second, numbers of M1 macrophages and proinflammatory cytokines are reduced in IFN-γR^−/−^ compared with WT controls (34). Besides, the treatment of SCI with anti-Vγ4 antibodies has a beneficial effect, similar to that obtained with anti-TNF-α (34). In conclusion, manipulation of γδ T cell functions may be a potential treatment approach for future CNS traumatic diseases.

### Other Central Nervous System Diseases

The pathological and clinical outcome of CNS diseases can also be affected by the intestinal microflora in the context of autoimmunity (120–124). This relationship has been particularly well established for the response to bacteria, including pathogens and commensals, within the intestinal compartment and its effects on the CNS, a connection that was recently termed the gut-brain axis. The gut environment has been found to significantly influence CNS diseases such as MS, EAE, and ischemic brain injury; however, immune cell mechanisms are unclear. In addition, the pathogenesis of intractable epilepsy is related to γδ T cells, where proinflammatory γδ T cells were concentrated in epileptogenic lesions, and their numbers positively associated with disease severity (125–127).

## Conclusion

Since the identification of γδ T cells, there has been a boom in related studies and discoveries. Equipped with functions of both innate and adaptive immune cells, γδ T cells can provide consequential functions in the development of CNS diseases, such as recognizing a diverse array of antigens, rapid production of inflammatory mediators, and influencing the differentiation of their αβ counterparts. Recently, understanding the inflammatory and immune roles of γδ T cells has resulted in the development of many prospective therapies for CNS diseases. However, the exact mechanisms behind their contributions are yet to be fully elucidated.

The pandemic of 2019 coronavirus disease COVID-19, caused by the SARS-CoV-2 virus infection, has caused worldwide mortality (128). Past pandemics have demonstrated that COVID-19 is accompanied by diverse neuropsychiatric symptoms, such as encephalopathy, neuromuscular dysfunction, or demyelinating processes (129). Whether recovered SARS-CoV-2 patients will exhibit an increased incidence of MS symptomatology or other delayed neurologic sequelae, is an important, yet unanswered. Nevertheless, more substantial shreds of evidence are required on different subtypes of γδ T cells for defining their opposing roles in CNS inflammation and explaining the confounding findings on their pathogenic or protective role in CNS diseases. In summary, this review discusses recent notable studies of the neuro-inflammatory and immune functions of γδ T cells, intending to understand their roles in CNS disease, which may be crucial for the effective immunotherapies.

## Author Contributions

JW and FZ contributed to editing the manuscript. ZL and CS provided administrative support. GS and WZ helped the manuscript editing and discussions. All authors contributed to the article and approved the submitted version.

## Funding

This work was supported by the National Natural Science Foundation of China (No. 31970862), the Natural Science Foundation of Guangdong Province (Nos. 2018A030313576, 2019A1515011335), and the Science and Technology Program of Guangzhou (No. 201803010001).

## Conflict of Interest

CS was employed by Lunan Pharmaceutical Group Co. Ltd.

The remaining authors declare that the research was conducted in the absence of any commercial or financial relationships that could be construed as a potential conflict of interest.
